# Scotopic thresholds on dark-adapted chromatic perimetry in healthy aging and age-related macular degeneration

**DOI:** 10.1038/s41598-021-89677-4

**Published:** 2021-05-14

**Authors:** Manjot Kaur Grewal, Shruti Chandra, Alan Bird, Glen Jeffery, Sobha Sivaprasad

**Affiliations:** 1 Institute of Ophthalmology, University College London, London, EC1V 9EL UK; 2 NIHR Moorfields Biomedical Research Centre, Moorfields Eye Hospital, London, UK

**Keywords:** Macular degeneration, Biomarkers

## Abstract

To evaluate the effect of aging, intra- and intersession repeatability and regional scotopic sensitivities in healthy and age-related macular degeneration (AMD) eyes. Intra- and intersession agreement and effect of age was measured in healthy individuals. The mean sensitivity (MS) and pointwise retinal sensitivities (PWS) within the central 24° with 505 nm (cyan) and 625 nm (red) stimuli were evaluated in 50 individuals (11 healthy and 39 AMD eyes). The overall intra- and intersession had excellent reliability (intraclass correlation coefficient, ICC > 0.90) and tests were highly correlated (Spearman r_s_ = 0.75–0.86). Eyes with subretinal drusenoid deposit (SDD) had reduced PWS centrally, particularly at inferior and nasal retinal locations compared with controls and intermediate AMD (iAMD) without SDD. There was no difference in MS or PWS at any retinal location between iAMD without SDD and healthy individuals nor between iAMD with SDD and non-foveal atrophic AMD groups. Eyes with SDD have reduced rod function compared to iAMD without SDD and healthy eyes, but similar to eyes with non-foveal atrophy. Our results highlight rod dysfunction is not directly correlated with drusen load and SDD location.

## Introduction

Age-related macular degeneration (AMD) is broadly classified into early, intermediate and late AMD based on retinal features such as the presence of drusen (including its size, number and location), pigmentary abnormalities and atrophy or choroidal neovascularization on colour photographs^1,2^. However, a multimodal imaging approach has clearly identified another phenotype called reticular pseudodrusen also referred to as subretinal drusenoid deposit (SDD) that may exist independently or with drusen in AMD^3–6^. This entity is associated with rod photoreceptor dysfunction^7–11^.

Histological evidence of preferential rod susceptibility in aging and AMD^12,13^ has been corroborated by several functional studies such as the rate of recovery of retinal sensitivity following exposure to a bright light, and by scotopic thresholds, which measure the minimum light that can be detected once the retina is fully adapted in the dark^14–22^.

Interpretation of earlier studies measuring scotopic sensitivity in aging is difficult due to different instrumentation and methodologies and the lack of clarity of age-related changes in scotopic thresholds^20,23,24^. A cross-sectional study by Jackson and Owsley reported a diffuse and gradual decrease of mean scotopic sensitivity (27 loci across central 18°) obtained on a modified Humphrey Field Analyser (HFA) by 0.08 log units per decade, a decline twice as fast compared with photopic sensitivity in otherwise healthy retinae^19^. Of interest, they found no variation in sensitivity by retinal eccentricity. Evidence also indicates that scotopic thresholds are further reduced in individuals with AMD particularly in the parafovea compared to age-matched controls^14,25^. However, some AMD individuals with varying severity retain normal scotopic function^14^.

Standard automated perimetry plays a central role in the assessment of scotopic function of individuals with AMD. It is most commonly measured with scotopic microperimetry using the scotopic Nidek microperimeter (MP-1S; Nidek Technologies, Padova, Italy) and the scotopic Macular Integrity Assessment (S-MAIA; CenterVue, Padova, Italy) with the in-built 4-2 staircase strategy^14,17,26–28^. Although the S-MAIA has an improved dynamic range over the MP-1S, both of these microperimeters have a limited dynamic range of luminance implicating floor and ceiling effects^28^. More recently, a dark-adapted chromatic perimeter (DAC) was developed (Medmont Pty Ltd International, Victoria, Australia), with large dynamic range (0–75 dB for cyan stimulus, 0–50 dB for red stimulus) with 135-fixed test locations covering a field up to 144° horizontally and 72° vertically. This also allows the ability to perform two-color dark-adapted perimetry in which sensitivity to cyan (505 nm) is compared with sensitivity to a red (625 nm) stimulus at the same location further quantifying the integrity of rod function^29^. The rods are highly sensitive to the 505 nm (cyan) stimuli by at least 2 log units more than cones and insensitive to 625 nm (red) stimuli. Therefore, a difference in thresholds between the cyan and red stimuli greater than 20 dB in dark-adapted healthy eyes is deemed to be rod mediated. A smaller difference between the two stimuli is indicative of rod dysfunction as the response is cone-mediated^29,30^. Previous studies using this device have shown reduced scotopic function in individuals with intermediate AMD, particularly in those with SDD, following dark adaptation for 20 min and using the 4-2 staircase strategy^10,11,31^. However, as this instrument does not have an in-built normative perimetric database, the effect of aging has only been evaluated in one study to our knowledge^30^. Although the test–retest repeatability has been previously published, it showed high variability of rod function^30,32,33^. We sought to investigate whether taking the mean of measurements and increasing decibel steps decreased this variability. Furthermore, only limited studies have examined spatial variation in scotopic function across AMD severity phenotypes and none included non-foveal atrophy stage.

The goals of the current study were to (1) investigate the effect of age, intrasession and intersession repeatability of scotopic thresholds from the DAC perimeter in healthy younger and older participants; (2) evaluate 2 dB step vs 3 dB step strategies; (3) investigate the effect of retinal location on scotopic sensitivity with cyan and red stimuli in healthy aging and in individuals with varying severity of AMD including SDD.

## Results

The inter grader and intra grader agreement of categorisation of the study participants in various disease severity levels were high (Cohen’s kappa coefficient = 0.96; Cohen’s kappa coefficient = 0.94 respectively).

### Test–retest variability in healthy aging

**Figure 1:**
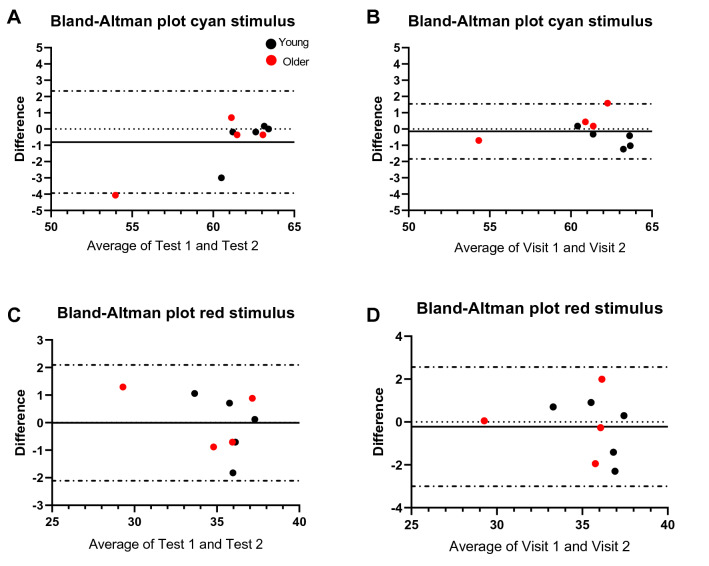
Bland–Altman plots with 95% limits of agreement (dashed lines) illustrate the difference in scotopic thresholds between intrasession measurements (**A**) cyan stimulus, (**C**) red stimulus, and between intersession measurements (**B**) cyan stimulus and (**D**) red stimulus. Black dotted line represents the mean between the two measurements. Black circles represent young participants (N = 5) and red circles represent older participants (N = 4).

**Figure 2:**
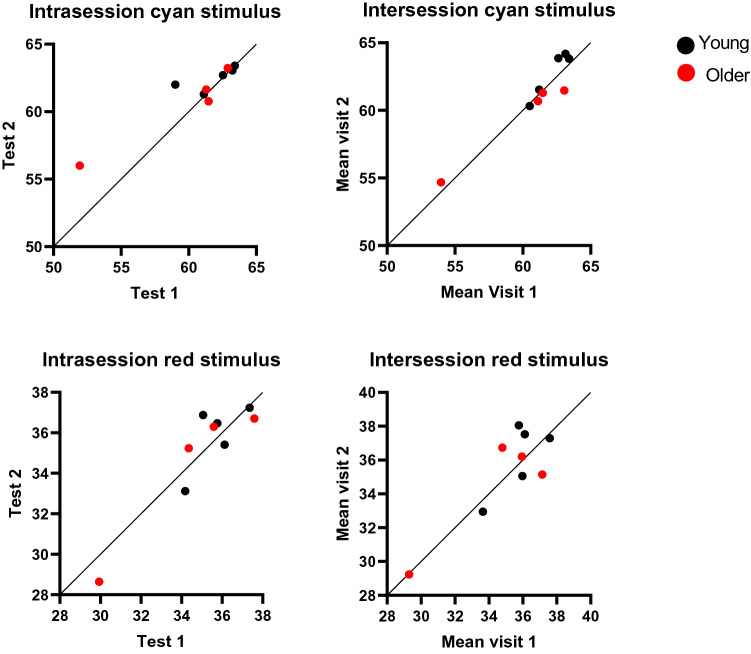
Scatter plots showing mean retinal sensitivity for both cyan and red stimuli of tests within one session and between two visits (young participants N = 5; older participants N = 4). The dots above the line of identity indicate improvement in the second session (test 2 or visit 2) and the dots below the line of identity indicate a decline in the second session.

A total of nine participants were recruited for the evaluation of repeatability for Medmont DAC perimeter; five younger (mean age (SD); 31.6 years (4.5), range 25.0–38.5 years) and four older participants (mean 61.0 years (2.5), range 59–64 years). Bland–Altman plots were generated to assess agreement between assessements shown in Fig. 1. Intraclass correlation coefficient (ICC) and Spearman correlation were calculated to quantify the observed agreement and is described in Table 1. The overall intrasession and intersession had excellent reliability (ICC > 0.90) and tests were highly correlated (Spearman r_s_ = 0.75–0.86). There was no definite indication of improvement in performance (learning effect) for both the cyan and red stimulus from the either first session (mean ± SD; 60.76 ± 3.58 dB and 35.10 ± 2.26 dB, respectively) to the second (61.57 ± 2.28 dB and 35.11 ± 2.73 dB, respectively) or from visit one (cyan 61.17 ± 2.89 dB; red 35.14 ± 2.48 dB) to visit two (cyan 61.31 ± 2.88 dB; red 35.36 ± 2.78 dB) as illustrated in Fig. 2. Therefore, scotopic thresholds measurement with Medmont DAC perimeter showed high reproducibility.

**Table 1:** Intraclass correlation coefficient (ICC) with 95% confidence intervals (CI), Spearman correlation (rho) and coefficient of repeatability (CoR) for mean sensitivity (MS) and pointwise sensitivity (PWS).

Outcome	ICC	Lower confidence limit	Upper confidence limit	Spearman correlation coefficient (r_s_)	p-value	CoR MS (dB)	CoR PWS (dB)
Intrasession (cyan)	0.91	0.64	0.98	0.83	0.008	3.35	5.96
Intersession (cyan)	0.98	0.91	0.995	0.85	0.006	1.62	4.47
Intrasession (red)	0.96	0.80	0.99	0.75	0.026	2.03	5.09
Intersession (red)	0.93	0.68	0.98	0.86	0.003	2.66	4.65
3 dB vs 2 dB cyan	0.86	0.47	0.96	0.77	0.012	–	–
3 dB vs 2 dB red	0.77	0.07	0.96	0.86	0.024	–	–

### Age-related change in retinal sensitivity

We assessed the change in retinal sensitivity with age in 20 healthy participants of various ages (mean 63.8 (± 15.4) years, range 25–77.2 years). Linear regression analysis revealed significant association between age and retinal sensitivity for the cyan stimulus (β coefficient = − 0.126, R^2^ = 0.302, p = 0.012) whereas no statistically significant association was found for the red stimulus (β coefficient = − 0.088, R^2^ = 0.191, p = 0.054).

### Scotopic thresholds reliability between 2 dB and 3 dB step strategies

**Figure 3:**
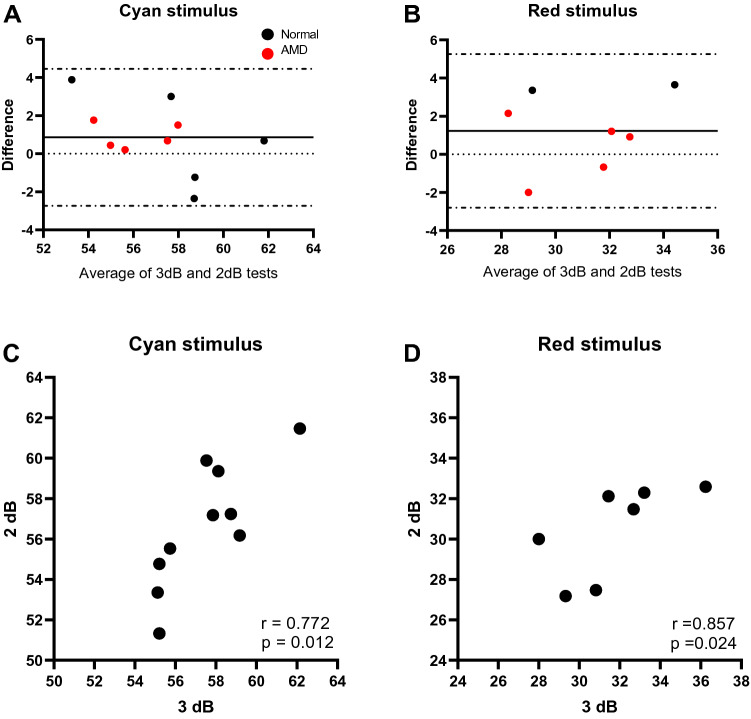
Bland–Altman plots with 95% limits of agreement (dashed lines) illustrates the difference in mean scotopic thresholds between 3 and 2 dB test strategies for both cyan (**A**) and red stimuli (**B**). Black dotted line represents the mean between the two measurements. Black circles represent healthy participants (N = 5 for cyan stimulus and N = 2 for red stimulus due to mechanical problems with the instrument) and red circles represent iAMD participants (N = 5 for both stimuli). The bottom two graphs show the correlations between 3 and 2 dB stimulus for the cyan (**C**) and red (**D**) stimuli.

Five healthy controls and five AMD participants (four individuals with iAMD no SDD and one with iAMD with SDD) completed these tests. The reliability between the two strategies was assessed with Bland–Altman plots which revealed good agreement between both strategies. On average, 2 dB step strategy measured 0.86 dB less than 3 dB step strategy for the cyan stimulus and 1.23 dB less for the red stimulus. Both strategies were strongly correlated for the cyan (r_s_ = 0.77, p = 0.012) and red (r_s_ = 0.86, p = 0.024) stimuli as displayed in Fig. 3. Intraclass correlation coefficient indicated good reliability (ICC > 0.75). These accuracy and reliability estimates are summarised in Table 1. The mean testing time for 2 dB and 3 dB steps for the cyan stimulus in healthy participants was 2.55 and 2.46 min respectively (p = 0.576) and in AMD individuals, 2.13 and 1.95 min respectively (p = 0.066). However, tests performed with red stimulus were significantly longer with 2 dB step than 3 dB step in the AMD group at an average test time of 2.19 and 1.95 min respectively (p = 0.042). No statistical analysis was performed in the healthy participants for the red stimulus as only data for two participants was available.

### Retinal sensitivity in healthy aging and varying AMD severity groups

There was no statistically significant difference between neither healthy controls and iAMD with no SDD nor between individuals with SDD and those with non-foveal atrophy. Mean retinal sensitivity measured with cyan and red stimuli was significantly reduced in the non foveal atrophic AMD group compared with healthy aging participants (cyan, p = 0.003; red, p = 0.0004) and compared to those with AMD no SDD (cyan, p = 0.0008; red, p = 0.002). Individuals with SDD had reduced rod function compared with healthy participants for both stimuli (cyan, p = 0.014; red, p = 0.008).

**Figure 4:**
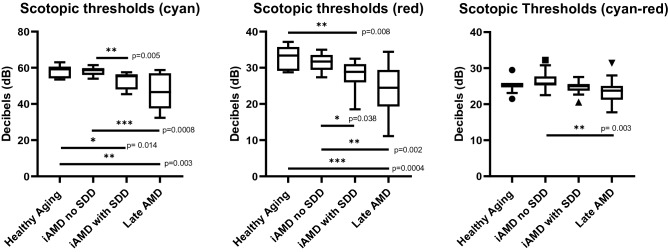
Boxplots for scotopic thresholds for cyan, red and difference between the two showing distribution of data across graded AMD severities. Black circles, squares and triangles show outliers (where data exceeds distance from the median by 1.5 times the interquartile range). Only significant differences between groups (P < 0.05) are displayed.

Participants with SDD also had lower thresholds when compared with iAMD withoutSDD (cyan, p = 0.005; red, p = 0.038). The difference between both stimuli was only statistically different between iAMD no SDD and participants with non-foveal atrophy (p = 0.003). Significant differences in mean retinal scotopic thresholds are illustrated in Fig. 4 for both stimuli.

**Figure 5:**
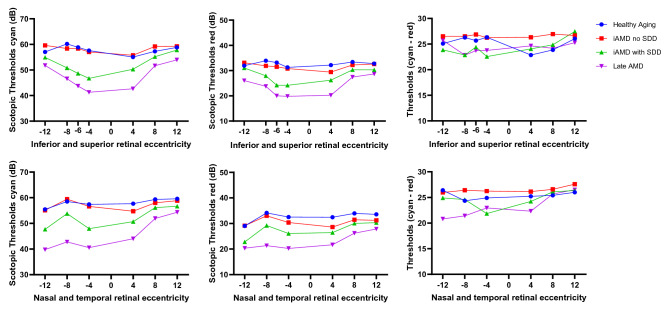
Scotopic thresholds thresholds at 4°, 8° and 12° eccentricity superior, inferior, temporal and nasal to the fovea) plus one further location at 6° inferior in the vertical meridian are shown for cyan and red stimulus. Four additional locations covering 12° eccentricities in all the four retinal quadrants not shown for illustrative purposes. The panels on the right show the difference between cyan and red thresholds. Negative eccentricities correspond to the inferior and nasal retina, positive eccentrcities to the superior and temporal retina.Healthy aging (N = 11), iAMD no SDD (N = 17), iAMD with SDD (N = 11) and Late AMD (N = 11).

Scotopic responses at specific loci for each AMD severity group are shown graphically in Fig. 5 and described in Tables S1–S3 in “Supplement”. Using the principle of two-color dark-adapted perimetry, the range of threshold difference (cyan minus red) at each retinal location was calculated to determine whether the responses were mediated by rods, cones, or a mix of both. All data obtained was mediated by rods.

**Figure 6:**
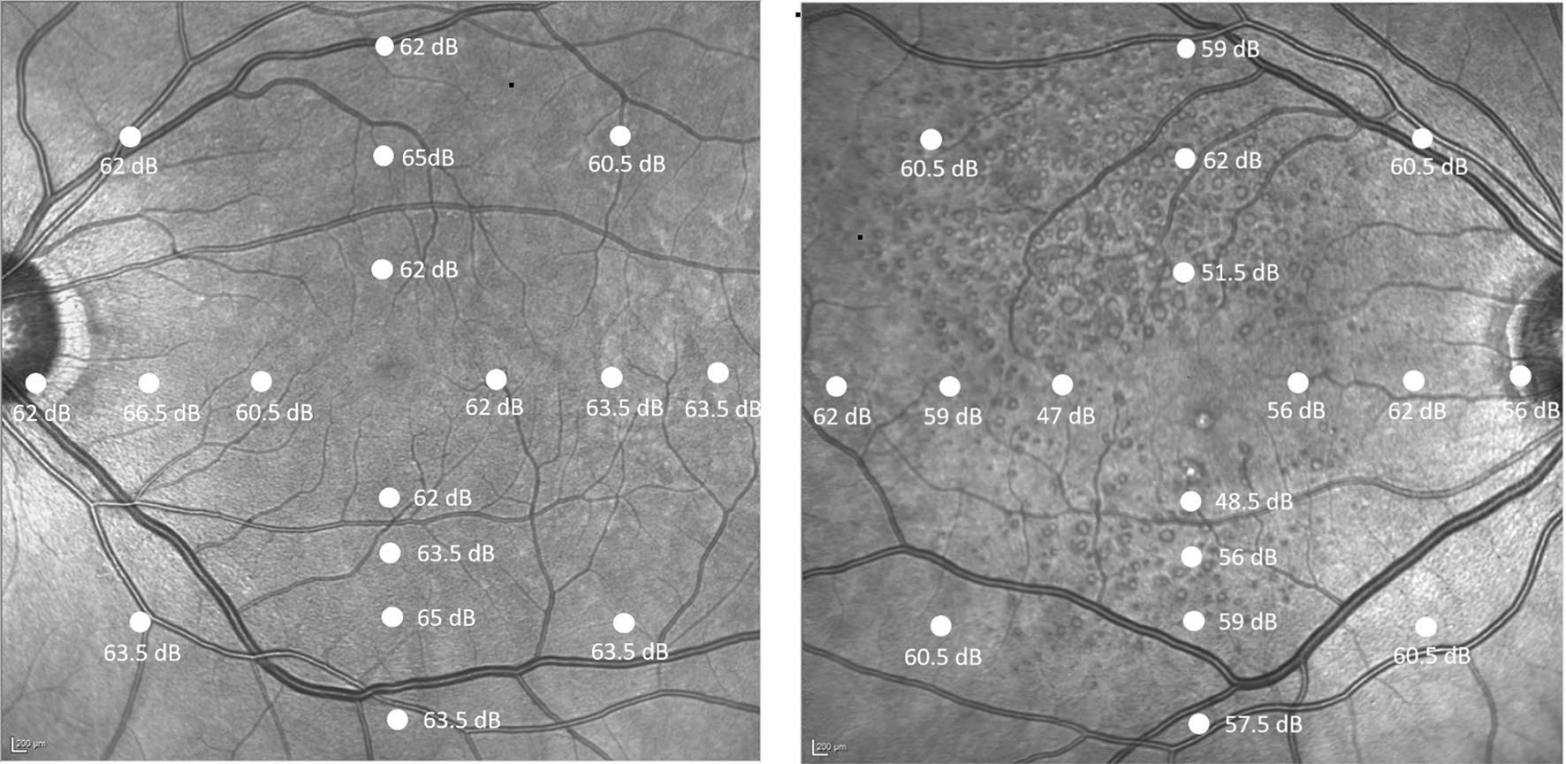
Example of pointwise sensitivities in a patient with healthy retina (**A**) and iAMD with SDD (**B**) with the scotopic thresholds topographically displayed over the infrared image across all 17 loci shown (4, 8, 12 and one additional location at 6° eccentricities nasal, temporal, superior and inferior to the fovea). The sensitivities were decreased in the paracentral loci compared to peripheral loci in (**B**) compared to (**A**). Images generated from Heidelberg Eye Explorer version 6.5).

There was no statistically significant difference between healthy participants and participants with iAMD without SDD nor between subjects with iAMD with SDD and late AMD at any retinal location for both cyan and red stimuli. Relative to healthy aging group, participants with non-foveal atrophy had reduced thresholds at all locations apart from more peripheral locations (8° and 12° superior retina, 12° temporal and supero-temporally). Similarly, individuals with advanced disease also had lower thresholds across all retinal locations compared to iAMD without SDD except for 4 locations (8° temporal; 12° temporal, superior and supero-nasal retinal eccentricities). Individuals with SDD had lower thresholds within the central 4° and more pronounced inferiorly, but no significant difference in the superior retina compared to healthy controls. A representative case is shown in Fig. 6. Relative to individuals with iAMD no SDD, participants with SDD had reduced rod function at inferior and nasal eccentricities and 12° supero temporal location, whereas no difference was found at temporal eccentricities. Pairwise comparisons between groups for all 17 retinal loci to both cyan and red stimuli with statistical significance are tabulated in Table S4 in “Supplement”.

## Discussion

This study demonstrated that it is feasible to utilize 3 dB-step strategy which is comparable to the commonly used 2 dB step strategy (4-2 staircase) with Medmont DAC perimeter. Both strategies were strongly correlated and ICC indicated good reliability. The mean scotopic thresholds obtained via 3 dB strategy (58.4 ± 2.5 dB) yielded comparable values to the 2 dB strategy for the healthy cohort reported by Bennett and colleagues (55.7 ± 4.7 dB) in older participants^30^. Although current perimetric strategies appear to be at near optimal levels, 3 dB strategy can reduce the mean number of trials (number of stimulus presentations) to reach optimal threshold, consequently reducing the testing time. Our results did not show statistically significant difference in the length of tests conducted between 2 and 3 dB for the cyan stimulus despite the trend of decreasing time with 3  dB step strategy. However, tests performed with 3 dB-step with the red stimulus were shorter than 2 dB step. Therefore, 3 dB test strategy is likely to be more efficient for wider retinal eccentricity testing as opposed to central macular testing without compromising accuracy.

The overall intrasession and intersession for mean retinal sensitivity had excellent reliability (ICC > 0.90), tests were highly correlated (Spearman r_s_ = 0.75–0.86) and there was no significant learning effect between sessions in our cohort in health aging. Our intrasession pointwise sensitivity CoR for both stimuli (cyan 5.96 dB and red 5.09 dB) was lower than the controls in the study by Tan and colleagues cyan: 8.4 dB, red: 6.7 dB) and comparable to the central PWS of 5.4 dB by Bennett and colleagues for the 505 nm stimulus^30,32^. Our CoR between visits (cyan: 4.47 dB, red: 4.65 dB) was lower than Tan and colleagues (cyan: 8.2 dB, red 6.2 dB) and by Bennett and colleagues (cyan: 6.0 dB) in our respective healthy cohorts^30,32^. It was also lower than the the overall CoR of 7.2 dB by Uddin et al. which comprised of three controls, four iAMD and five patients with SDD^33^. Therefore, taking the average of two of retinal sensitivity measurements between visits improves coefficient of repeatibility and the evaluation of scotopic sensitivity with Medmont DAC perimeter showed high reproducibility.

Our results also validated previous findings on the effect of aging in participants with normal retina on DAC perimetry. Bennett and colleagues found an overall decrease in scotopic thresholds of 1.2 dB in aging^30^. Similarly, we observed 1.26 dB decrease per decade.

The principle of two-color dark-adapted perimetry, which exploits the differential spectral sensitivities between rods and cones, was used to determine whether the responses were mediated by rods, cones, or a mix of both^34^. Based on this approach, all data obtained in our study was mediated by rods as the difference was greater than 20 dB. Our study also shows particularly reduced mean retinal sensitivity in individuals with SDD which is in concordance with previous studies^10,31^. Interestingly, we found no difference in mean scotopic sensitivity in individuals with iAMD without SDD when compared with healthy controls. This finding is substantiated by Tan et al., where they found that scotopic thresholds were indistinguishable between controls and iAMD without SDD when there was no preceding photobleach^31^. This is in contrast to other studies that have shown reduced thresholds following dark adaptation between these two groups^10,11^. However, this is likely due to prior exposure of pre-adapting light and shorter length of time of dark adaptation of 20 min in both of these studies whereas we used 40 min^11^. It can take 30–45 min for rods to reach to their maximum sensitivity for healthy dark-adapted eyes and therefore some AMD individuals may still have been dark adapting at 20 min^35^. In addition, the duration and intensity of the preadapting light affects the level of thresholds^36^. Therefore, the photobleach preceding scotopic thresholds measurements in other studies may have inadvertently affected absolute scotopic sensitivity. Hence, under optimal conditions, rod scotopic sensitivity in iAMD eyes is similar to healthy eyes.

Our data were also unable to elicit any difference in mean scotopic sensitivity between individuals with SDD and those with non-foveal atrophy, suggesting that the presence of SDD is an indicator of severe disease and the functional outcomes are as poor as those with non-foveal atrophy.

When we consider our analysis of pointwise sensitivities, there was an overall decrease in sensitivity for almost all locations for atrophic individuals compared with normal and individuals with iAMD without SDD, but no significant difference at any retinal loci when compared to individuals with AMD with SDD. Consistent with our mean retinal sensitivity results, there was no difference in pointwise sensitivity at any retinal location between iAMD without SDD and healthy controls. Importantly, these findings suggest that the overall loss of rod function is not directly correlated with drusen load or extent of GA.

Our results also revealed that individuals with SDD not only have reduced thresholds centrally but are more depressed at inferior and nasal retinal locations compared with controls and iAMD without SDD. This is an interesting finding, as SDD are usually more abundant in the superior perifovea^37^. In addition, scotopic thresholds were able to differentiate iAMD eyes with and without SDD in 11 out of 17 loci. Thus, we conclude that SDD in a structural sign of rod dysfunction which is independent of SDD location.

Strengths of our study include methodical and consistent procedures and validated retinal grading by two graders with multimodal imaging. Dark adaptation of 40 min allowed for rod photoreceptors to reach maximum sensitivity. We also included the non-foveal GA phenotype which has not been investigated in similar studies previously. The main limitation of our study is the small sample sizes for our groups. Despite this, statistically significant rod function deficits were found in SDD eyes comparable to non-foveal atrophic eyes. A larger AMD cohort and longitudinal analysis would further validate our findings.

In conclusion, our study demonstrated the feasibility of using 3 dB step protocol for measuring scotopic thresholds and using the mean of two tests reduced intrasession and intersession variability. Eyes with SDD have reduced rod function compared to iAMD without SDD and healthy eyes, but similar to eyes with non-foveal atrophy. Our results highlight rod dysfunction is not directly correlated with drusen load, SDD location or extent of GA.

## Methods

### Ethics approval

Participants were provided with an information sheet and informed consent was obtained from each participant. The study conformed to the standards set by the Declaration of Helsinki, and the procedures were approved by the Camden and Kings Cross NRES Committee London REC 16/LO/1317.

### Participants

Participants included in the studies were classified in groups as described below with colour fundus photography and confirmed using infrared and OCT modalities by 2 graders (M.G and S.C):

1. Healthy aging: includes those with either no retinal abnormalities or normal aging changes (drupelets) and early AMD as the latter has a low risk of conversion to late stages of AMD
2. iAMD no SDD: includes all individuals with more than one large drusen with or without any pigmentary changes. None of these patients had any evidence of geographic atrophy.
3. iAMD with SDD: , includes individuals with the presence of at least five macular SDD that have penetrated the ELM and disrupting the ellipsoid zone on Spectralis OCT confirmed by infrared reflectance (Spectralis OCT, Heidelberg Engineering, Germany). All of these patients except one, had all features of iAMD group with no SDD. None of these patients had any evidence of geographic atrophy.
4. Late AMD: comprises of individuals with non-foveal geographic atrophy secondary to dry age-related macular degeneration. Geographic atrophy was defined as any sharply delineated roughly round or oval area of hypopigmentation or depigmentation with increased visibility of the underlying choroidal vessels and of at least 175 mm in diameter on 30 or 35 CFP images^2^. On OCT, atrophic features were assessed according to CAM classification defined as complete RPE and outer retinal atrophy (cRORA) defined as a region of hypertransmission of at least 250 mm in diameter; a zone of attenuation or disruption of the RPE of at least 250 mm in diameter; evidence of overlying photoreceptor degeneration, and absence of scrolled RPE or other signs of an RPE tear^38^. None of these eyes had any evidence of neovascular AMD.

#### Exclusion criteria

Participants were excluded if there was co-existent ocular disease (neovascular AMD, glaucoma or diabetic retinopathy, substantial cataract) in the study eye, significant systemic disease or history of medication known to affect visual function, epilepsy, history of major ocular surgery in the last 3 months or anticipated within the next 6 months following enrolment in the study eye and any allergies to adhesives or any other component used.

### Procedure: medmont dark-adapted chromatic perimetry

Dark-adapted retinal sensitivity was measured with a Medmont DACP (Medmont Pty Ltd International, Victoria, Australia), which is a blackened bowl perimeter with 135-fixed test locations covering a field up to 144° horizontally and 72° vertically. In our study, the perimetric test grid consisted of 17 loci shown (4°, 8°, 12° eccentricities nasal, temporal, superior and inferior to the fovea with one additional location at 6° inferiorly). A single exam on this device take just over 2 min to perform.

Pupils of the individuals were dilated to at least 6 mm with 2.5% phenylephrine and 1% tropicamide. The participant was seated in the dark for 40 min with a black sleeping mask on their eyes to avoid any light reaching the eye. Appropriate corrective lenses were placed in the lens holder to account for participant’s refraction for a viewing distance of 30 cm. Individuals were instructed to focus on the central red fixation light at all times and to press the response button when a light stimulus was seen. Fixation was monitored using an infrared camera built in the perimeter. The light stimulus was 1.73° in size (Goldmann size V) and was presented for 110 ms in a random order across all retinal locations using 3 dB steps.

### Outcome assessments

1. Test–retest on healthy individuals.
  Intrassesion reliability consisted of nine healthy participants repeating the same assessment immediately following the first test with the cyan stimulus. This was followed by two consecutive assessments with the red stimulus. All tests were conducted at 3 dB steps. Measurements were repeated on a separate visit at approximately 7 days (+ 7 days) from the initial visit to determine intersession test–retest repeatability. Intersession reliability was measured as the agreement between the average of the two tests carried out at the first visit against the average of the two consecutive tests at the second visit for both stimuli.
2. Age-related change in retinal sensitivity.
  A total of 20 healthy participants of various ages (range 25–77.2 years) underwent the above test with 3 dB test strategy to evaluate the age-related changes in retinal sensivity. These included nine of the participants included in the test–retest cohort.
3. Comparison of 2 dB vs 3 dB testing strategy.
  The protocol was carried out with 3 dB step strategy and was repeated with 2 dB strategy either on the same day or within the next 2 days on five healthy age-matched individuals and five individuals with intermediate AMD.
4. Difference in scotopic threshold between cyan and red stimulus.
  A total of 50 individuals consisting of 11 healthy individuals and 39 AMD individuals with varying severity of AMD (iAMD no SDD N = 17, iAMD with SDD N = 11 and Late AMD N = 11) were evalauted to assess the difference in scotopic threshold between cyan and red stimulus. Following dark adaptation, scotopic thresholds were measured monocularly in the study eye with a 505 nm (cyan) stimulus at 17 retinal locations (4°, 8° and 12° eccentricity superior, inferior, temporal and nasal to the fovea) plus one additional location at 6° inferior in the vertical meridian and at four locations covering 12° eccentricities in all the four retinal quadrants. This was repeated with a 625 nm (red) stimulus. Scotopic thresholds were analysed as the mean retinal sensitivity from all 17 loci with with both cyan and red stimuli and further evaluated by a pointwise method to investigate wether a particular area of the retina was more vulnerable to scotopic dysfunction.

### Statistical analysis

Statistical analyses were performed with GraphPad Prism. The normal distribution of the data was verified using the Shapiro–Wilk test. To examine the intrasession and intersession repeatability and accuracy in healthy aging, Bland–altman plots, Intraclass correlation coefficient (ICC), Spearmans’s correlation coefficient (r_s_) were calculated. Coefficient of repeatability (CoR) was calculated as 1.96 times the standard deviation (SD) of the difference between the two measurements. The agreement between 2 and 3 dB-step was also analysed similarly. Linear regression analysis was performed to investigate the association between age and mean retinal sensitivity. Mean retinal sensitivity for both stimuli (and the difference between them) was calculated and compared among the study groups using Kruskal Wallis test and post hoc multiple comparisons Dunn’s uncorrected test. To evaluate the extent of rod dysfunction as a function of eccentricity, the point-wise sensitivity for each test point were calculated. The significance level was set at p < 0.05. The inter grader and intra grader agreement was measured using Cohen’s kappa coefficient.

## Supplementary Information

Supplementary Tables.

## References

[1] Ferris FL (2013). Clinical classification of age-related macular degeneration. Ophthalmology.

[2] Bird AC (1995). An international classification and grading system for age-related maculopathy and age-related macular degeneration. The International ARM Epidemiological Study Group. Surv. Ophthalmol..

[3] Finger RP (2016). Reticular pseudodrusen and their association with age-related macular degeneration: The Melbourne collaborative cohort study. Ophthalmology.

[4] Wu Z, Ayton LN, Luu CD, Baird PN, Guymer RH (2016). Reticular pseudodrusen in intermediate age-related macular degeneration: Prevalence, detection, clinical, environmental, and genetic associations. Invest. Ophthalmol. Vis. Sci..

[5] Steinberg JS, Auge J, Fleckenstein M, Holz FG, Schmitz-Valckenberg S (2015). Longitudinal analysis of reticular drusen associated with age-related macular degeneration using combined confocal scanning laser ophthalmoscopy and spectral-domain optical coherence tomography imaging. Ophthalmologica.

[6] Cozzi M (2020). Sensitivity and specificity of multimodal imaging in characterizing Drusen. Ophthalmol. Retina.

[7] Grewal MK (2020). Exploratory study on visual acuity and patient-perceived visual function in patients with subretinal drusenoid deposits. J. Clin. Med..

[8] Flamendorf J (2015). Impairments in dark adaptation are associated with age-related macular degeneration severity and reticular pseudodrusen. Ophthalmology.

[9] Dimitrov PN, Guymer RH, Zele AJ, Anderson AJ, Vingrys AJ (2008). Measuring rod and cone dynamics in age-related maculopathy. Invest. Ophthalmol. Vis. Sci..

[10] Flynn OJ, Cukras CA, Jeffrey BG (2018). Characterization of rod function phenotypes across a range of age-related macular degeneration severities and subretinal drusenoid deposits. Invest. Ophthalmol. Vis. Sci..

[11] Fraser RG (2016). Assessment of retinotopic rod photoreceptor function using a dark-adapted chromatic perimeter in intermediate age-related macular degeneration. Invest. Ophthalmol. Vis. Sci..

[12] Curcio CA, Millican CL, Allen KA, Kalina RE (1993). Aging of the human photoreceptor mosaic: Evidence for selective vulnerability of rods in central retina. Invest. Ophthalmol. Vis. Sci..

[13] Curcio CA, Medeiros NE, Millican CL (1996). Photoreceptor loss in age-related macular degeneration. Invest. Ophthalmol. Vis. Sci..

[14] Owsley C (2000). Psychophysical evidence for rod vulnerability in age-related macular degeneration. Invest. Ophthalmol. Vis. Sci..

[15] Owsley C, Jackson GR, White M, Feist R, Edwards D (2001). Delays in rod-mediated dark adaptation in early age-related maculopathy. Ophthalmology.

[16] Owsley C (2014). Associations between abnormal rod-mediated dark adaptation and health and functioning in older adults with normal macular health. Invest. Ophthalmol. Vis. Sci..

[17] Jackson GR, Owsley C, Cordle EP, Finley CD (1998). Aging and scotopic sensitivity. Vision Res..

[18] Jackson GR, Owsley C, McGwin G (1999). Aging and dark adaptation. Vision Res..

[19] Jackson GR, Owsley C (2000). Scotopic sensitivity during adulthood. Vision Res..

[20] Sturr JF, Zhang L, Taub HA, Hannon DJ, Jackowski MM (1997). Psychophysical evidence for losses in rod sensitivity in the aging visual system. Vision Res..

[21] Luu CD (2018). Topographic rod recovery profiles after a prolonged dark adaptation in subjects with reticular pseudodrusen. Ophthalmol. Retina.

[22] Chen KG (2019). Longitudinal study of dark adaptation as a functional outcome measure for age-related macular degeneration. Ophthalmology.

[23] Pulos E (1989). Changes in rod sensitivity through adulthood. Invest. Ophthalmol. Vis. Sci..

[24] Hammond BR (1998). Scotopic sensitivity: Relation to age, dietary patterns, and smoking status. Optom. Vis. Sci..

[25] Steinmetz RL, Haimovici R, Jubb C, Fitzke FW, Bird AC (1993). Symptomatic abnormalities of dark adaptation in patients with age-related Bruch's membrane change. Br. J. Ophthalmol..

[26] Crossland MD, Luong VA, Rubin GS, Fitzke FW (2011). Retinal specific measurement of dark-adapted visual function: Validation of a modified microperimeter. BMC Ophthalmol..

[27] Nebbioso M, Barbato A, Pescosolido N (2014). Scotopic microperimetry in the early diagnosis of age-related macular degeneration: Preliminary study. Biomed. Res. Int..

[28] Steinberg JS (2017). Evaluation of two systems for fundus-controlled scotopic and mesopic perimetry in eye with age-related macular degeneration. Transl. Vis. Sci. Technol..

[29] Bennett LD, Klein M, Locke KG, Kiser K, Birch DG (2017). Dark-adapted chromatic perimetry for measuring rod visual fields in patients with retinitis pigmentosa. Transl. Vis. Sci. Technol..

[30] Bennett LD (2019). Regional variations and intra-/intersession repeatability for scotopic sensitivity in normal controls and patients with inherited retinal degenerations. Invest. Ophthalmol. Vis. Sci..

[31] Tan R, Guymer RH, Luu CD (2018). Subretinal drusenoid deposits and the loss of rod function in intermediate age-related macular degeneration. Invest. Ophthalmol. Vis. Sci..

[32] Tan RS, Guymer RH, Luu CD (2018). Repeatability of retinal sensitivity measurements using a medmont dark-adapted chromatic perimeter in healthy and age-related macular degeneration cases. Transl. Vis. Sci. Technol..

[33] Uddin D (2020). Repeatability of scotopic sensitivity and dark adaptation using a medmont dark-adapted chromatic perimeter in age-related macular degeneration. Transl. Vis. Sci. Technol..

[34] Jacobson, S. G., Apathy, P. P. & Parel, J.-M. Rod and cone perimetry: computerized testing and analysis. In *Principles and Practice of Clinical Electrophysiology of Vision* (eds Heckenlively, J. R. and Arden, G. B.) Ch. 60, 475–482 (Mosby-Year Book, Inc, 1991).

[35] Tipton DA (1984). A review of vision physiology. Aviat. Space Environ. Med..

[36] Hecht S, Haig C, Wald G (1935). The dark adaptation of retinal fields of different size and location. J. Gen. Physiol..

[37] Curcio CA (2013). Subretinal drusenoid deposits in non-neovascular age-related macular degeneration: Morphology, prevalence, topography, and biogenesis model. Retina.

[38] Sadda SR (2018). Consensus definition for atrophy associated with age-related macular degeneration on OCT: Classification of atrophy report 3. Ophthalmology.

