# Supplementary material for: Scotopic thresholds on dark-adapted chromatic perimetry in healthy aging and age-related macular degeneration
Source: Sci Rep. 2021 May 14;11:10349. doi: 10.1038/s41598-021-89677-4 (PMC8121851; doi:10.1038/s41598-021-89677-4)
Supplement: Supplementary file 1 — Supplementary Tables. [file 41598_2021_89677_MOESM1_ESM.docx]

**Scotopic thresholds on dark-adapted chromatic perimetry in healthy ageing and age-related macular degeneration**

Manjot Kaur Grewal^1, 2^, Shruti Chandra^1, 2^, Alan Bird^1^, Glen Jeffery^1^, and Sobha Sivaprasad^1, 2*^

^1^University College London, Institute of Ophthalmology, London, EC1V 9EL, UK

^2^ NIHR Moorfields Biomedical Research Centre, Moorfields Eye Hospital, London, UK

^*^Corresponding author: sobha.sivaprasad@nhs.net

Supplement

*Table 1. Mean scotopic thresholds to cyan stimuli across all retinal eccentricities per AMD severity group.*

|  | Group, Mean ± SD | | | | |  | |
| --- | --- | --- | --- | --- | --- | --- | --- |
| Retinal eccentricity | | **Healthy aging (N = 11)** | **iAMD no SDD (N = 17)** | **iAMD with SDD**  **(N = 11)** | **Late AMD**  **(N = 11)** | |  |
| 4° superior | | 55.05 ± 5.58 | 55.74 ± 6.05 | 50.27 ± 6.07 | 42.64 ± 14.93 | |  |
| 8° superior | | 57.27 ± 4.82 | 59.18 ± 3.09 | 55.18 ± 4.99 | 51.64 ± 7.60 | |  |
| 12° superior | | 58.82 ± 4.03 | 59.26 ± 3.98 | 57.77 ± 4.29 | 53.95 ± 9.42 | |  |
| 12° supero-nasal | | 58.14 ± 3.91 | 59.53 ± 2.85 | 55.86 ± 5.75 | 53.68 ± 9.45 | |  |
| 12° supero-temporal | | 58.86 ± 3.11 | 59.71 ± 2.49 | 55.73 ± 5.27 | 56.27 ± 4.18 | |  |
| 4° inferior | | 57.59 ± 5.51 | 57.06 ± 2.64 | 46.73 ± 7.28 | 41.27 ± 13.36 | |  |
| 6° inferior | | 58.86 ± 4.80 | 58.38 ± 3.19 | 48.64 ± 7.24 | 43.73 ± 11.75 | |  |
| 8° inferior | | 60.18 ± 4.94 | 58.38 ± 2.49 | 50.82 ± 8.20 | 46.59 ± 11.04 | |  |
| 12° inferior | | 57.05 ± 8.89 | 59.62 ± 2.49 | 54.91 ± 4.45 | 51.77 ± 8.45 | |  |
| 12° infero-nasal | | 57.59 ± 4.64 | 58.82 ± 2.54 | 54.09 ± 4.75 | 46.05 ±12.28 | |  |
| 12° infero-temporal | | 58.05 ± 4.32 | 58.56 ± 3.08 | 54.77 ± 5.10 | 51.50 ± 7.53 | |  |
| 4° nasal | | 57.41 ± 4.04 | 56.62 ± 4.08 | 47.95 ± 9.66 | 40.55 ± 20.90 | |  |
| 8° nasal | | 58.50 ± 4.60 | 59.44 ± 2.84 | 53.82 ± 7.21 | 42.77 ± 15.04 | |  |
| 12° nasal | | 55.45 ± 5.56 | 55.12 ± 4.69 | 47.68 ± 8.36 | 39.77 ± 11.92 | |  |
| 4° temporal | | 57.68 ± 5.67 | 54.76 ± 6.26 | 50.68 ± 8.73 | 44.00 ± 12.35 | |  |
| 8° temporal | | 59.36 ± 3.96 | 58.03 ± 2.48 | 56.14 ± 4.42 | 51.91 ± 8.51 | |  |
| 12° temporal | | 59.59 ± 3.56 | 58.85 ± 2.63 | 56.68 ± 5.59 | 54.36 ± 8.60 | |  |

*Table 2. Mean scotopic thresholds to red stimuli across all retinal eccentricities per AMD severity group.*

|  | Group, Mean ± SD | | | | |  | |
| --- | --- | --- | --- | --- | --- | --- | --- |
| Retinal eccentricity | | **Healthy aging (N = 11)** | **iAMD no SDD (N = 17)** | **iAMD with SDD**  **(N = 11)** | **Late AMD**  **(N = 11)** | |  |
| 4° superior | | 32.18 ± 3.39 | 29.41 ± 5.77 | 26.23 ± 3.47 | 20.25 ± 8.13 | |  |
| 8° superior | | 33.41 ± 3.75 | 32.24 ± 3.53 | 30.32 ± 2.80 | 27.45 ± 6.30 | |  |
| 12° superior | | 32.77 ± 3.34 | 32.59 ± 2.93 | 30.32 ± 3.70 | 28.68 ± 5.67 | |  |
| 12° supero-nasal | | 32.68 ± 3.45 | 33.12 ± 2.32 | 30.82 ± 4.42 | 28.45 ± 5.95 | |  |
| 12° supero-temporal | | 32.64 ± 3.64 | 33.38 ± 1.68 | 30.18 ± 4.52 | 29.09 ± 5.20 | |  |
| 4° inferior | | 31.27 ± 3.90 | 30.82 ± 3.51 | 24.18 ± 6.13 | 19.80 ± 10.66 | |  |
| 6° inferior | | 33.18 ± 4.81 | 31.53 ± 3.75 | 24.23 ± 8.87 | 20.00 ± 10.69 | |  |
| 8° inferior | | 33.91 ± 4.61 | 31.88 ± 3.94 | 27.95 ± 6.31 | 23.77 ± 8.29 | |  |
| 12° inferior | | 31.95 ± 8.00 | 33.12 ± 4.25 | 31.05 ± 4.33 | 26.05 ± 8.88 | |  |
| 12° infero-nasal | | 33.73 ± 4.34 | 31.44 ± 3.74 | 28.95 ± 5.94 | 23.50 ± 8.93 | |  |
| 12° infero-temporal | | 33.05 ± 4.04 | 31.97 ± 3.60 | 29.41 ± 5.44 | 26.41 ± 7.65 | |  |
| 4° nasal | | 32.50 ± 3.22 | 30.38 ± 2.19 | 26.09 ± 5.70 | 20.25 ± 11.56 | |  |
| 8° nasal | | 34.14 ± 3.18 | 33.03 ± 2.54 | 29.23 ± 5.96 | 21.36 ± 10.92 | |  |
| 12° nasal | | 29.05 ± 5.48 | 29.15 ± 4.45 | 22.77 ± 7.75 | 20.35 ± 6.54 | |  |
| 4° temporal | | 32.45 ± 3.88 | 28.62 ± 4.89 | 26.45 ± 4.96 | 21.68 ± 9.72 | |  |
| 8° temporal | | 33.95 ± 4.37 | 31.44 ± 3.78 | 30.05 ± 4.04 | 26.23 ± 8.24 | |  |
| 12° temporal | | 33.59 ± 4.30 | 31.26 ± 3.61 | 30.32 ± 3.99 | 27.86 ± 7.69 | |  |

*Table 3. Mean scotopic thresholds difference (cyan -red) stimuli across all retinal eccentricities per AMD severity group.*

|  | Group, Mean ± SD | | | | |  | |
| --- | --- | --- | --- | --- | --- | --- | --- |
| Retinal eccentricity | | **Healthy aging (N = 11)** | **iAMD no SDD (N = 17)** | **iAMD with SDD**  **(N = 11)** | **Late AMD**  **(N = 11)** | |  |
| 4° superior | | 22.86 ± 4.20 | 26.32 ± 3.71 | 24.05 ± 4.80 | 24.65 ± 10.30 | |  |
| 8° superior | | 23.86 ± 4.50 | 26.94 ± 3.22 | 24.86 ± 5.43 | 24.18 ± 3.31 | |  |
| 12° superior | | 26.05 ± 4.06 | 26.68 ± 3.22 | 27.45 ± 3.69 | 25.27 ± 5.88 | |  |
| 12° supero-nasal | | 25.45 ± 3.83 | 26.41 ± 2.02 | 25.05 ± 3.42 | 25.23 ± 4.80 | |  |
| 12° supero-temporal | | 26.23 ± 3.13 | 26.32 ± 2.65 | 25.55 ± 5.62 | 27.18 ± 4.81 | |  |
| 4° inferior | | 26.32 ± 4.07 | 26.24 ± 3.94 | 22.55 ± 2.79 | 23.75 ± 5.81 | |  |
| 6° inferior | | 25.68 ± 3.70 | 26.85 ± 2.93 | 24.41 ± 5.40 | 23.73 ± 5.69 | |  |
| 8° inferior | | 26.27 ± 2.81 | 26.50 ± 2.90 | 22.86 ± 2.28 | 22.82 ± 4.71 | |  |
| 12° inferior | | 25.09 ± 2.81 | 26.50 ± 4.53 | 23.86 ± 2.84 | 25.73 ± 3.19 | |  |
| 12° infero-nasal | | 23.86 ± 2.65 | 27.38 ± 3.40 | 25.14 ± 4.27 | 22.55 ± 5.70 | |  |
| 12° infero-temporal | | 25.00 ± 2.58 | 26.59 ± 2.34 | 25.36 ± 3.32 | 25.09 ± 1.67 | |  |
| 4° nasal | | 24.91 ± 2.21 | 26.24 ± 3.32 | 21.86 ± 5.22 | 22.95 ± 11.77 | |  |
| 8° nasal | | 24.36 ± 3.86 | 26.41 ± 2.98 | 24.59 ± 4.40 | 21.41 ± 7.13 | |  |
| 12° nasal | | 26.41 ± 6.58 | 25.97 ± 3.35 | 24.91 ± 3.16 | 20.80 ± 7.48 | |  |
| 4° temporal | | 25.23 ± 4.30 | 26.15 ± 5.97 | 24.23 ± 7.34 | 22.32 ± 7.60 | |  |
| 8° temporal | | 25.41 ± 4.03 | 26.59 ± 3.65 | 26.09 ± 2.43 | 25.68 ± 5.30 | |  |
| 12° temporal | | 26.00 ± 2.83 | 27.59 ± 3.42 | 26.36 ± 3.51 | 26.50 ± 4.24 | |  |

*Table 4. Pairwise comparisons between groups for all 17 retinal loci to both cyan and red stimuli (unpaired, non-parametric Kruskal Wallis test with post hoc Dunn’s uncorrected test)*

| Retinal eccentricity | Stimuli | Healthy aging vs iAMD no SDD | Healthy aging vs iAMD with SDD | Healthy aging vs Late AMD | iAMD no SDD vs iAMD with SDD | iAMD no SDD vs Late AMD | iAMD with SDD vs Late AMD |
| --- | --- | --- | --- | --- | --- | --- | --- |
| 4° superior | cyan | 0.708 | 0.111 | **0.039** | **0.033** | **0.008** | 0.634 |
|  | red | 0.197 | **0.005** | **0.0002** | 0.067 | **0.004** | 0.309 |
| 8° superior | cyan | 0.293 | 0.382 | 0.095 | **0.044** | **0.004** | 0.427 |
|  | red | 0.467 | **0.029** | **0.007** | 0.094 | **0.025** | 0.604 |
| 12° superior | cyan | 0.594 | 0.797 | 0.208 | 0.414 | 0.055 | 0.316 |
|  | red | 0.972 | 0.171 | **0.048** | 0.123 | **0.027** | 0.544 |
| 12° supero-nasal | cyan | 0.476 | 0.365 | 0.272 | 0.087 | 0.055 | 0.848 |
|  | red | 0.745 | 0.302 | **0.034** | 0.144 | **0.008** | 0.275 |
| 12° supero-temporal | cyan | 0.517 | 0.160 | 0.156 | **0.028** | **0.027** | 0.988 |
|  | red | 0.487 | 0.220 | 0.157 | **0.041** | **0.024** | 0.852 |
| 4° inferior | cyan | 0.870 | **0.001** | **0.0003** | **0.0005** | **0.0001** | 0.725 |
|  | red | 0.900 | **0.006** | **0.002** | **0.004** | **0.001** | 0.707 |
| 6° inferior | cyan | 0.793 | **0.001** | **0.0004** | **0.0009** | **0.0003** | 0.752 |
|  | red | 0.437 | **0.004** | **0.0005** | **0.016** | **0.002** | 0.572 |
| 8° inferior | cyan | 0.600 | **0.004** | **0.0006** | **0.009** | **0.001** | 0.552 |
|  | red | 0.242 | **0.011** | **0.0006** | **0.010** | **0.010** | 0.390 |
| 12° inferior | cyan | 0.572 | 0.056 | **0.026** | **0.008** | **0.003** | 0.746 |
|  | red | 0.879 | 0.223 | **0.024** | 0.234 | **0.020** | 0.298 |
| 12° infero-nasal | cyan | 0.593 | 0.085 | **0.006** | **0.015** | **0.0003** | 0.293 |
|  | red | 0.210 | **0.047** | **0.0006** | 0.351 | **0.011** | 0.146 |
| 12° infero-temporal | cyan | 0.627 | 0.181 | **0.029** | 0.050 | **0.004** | 0.398 |
|  | red | 0.485 | 0.084 | **0.010** | 0.228 | **0.034** | 0.404 |
| 4° nasal | cyan | 0.533 | **0.008** | **0.011** | **0.022** | **0.029** | 0.918 |
|  | red | 0.135 | **0.002** | **0.0006** | 0.054 | **0.020** | 0.682 |
| 8 °nasal | cyan | 0.503 | 0.086 | **0.004** | **0.010** | **0.0001** | 0.240 |
|  | red | 0.410 | **0.012** | **0.0001** | 0.052 | **0.0008** | 0.196 |
| 12° nasal | cyan | 0.950 | 0.071 | **0.002** | **0.040** | **0.0005** | 0.189 |
|  | red | 0.851 | **0.030** | **0.002** | **0.028** | **0.002** | 0.347 |
| 4° temporal | cyan | 0.301 | **0.035** | **0.002** | 0.197 | **0.017** | 0.323 |
|  | red | 0.074 | **0.009** | **0.0004** | 0.273 | **0.032** | 0.344 |
| 8° temporal | cyan | 0.315 | 0.090 | **0.009** | 0.389 | 0.059 | 0.353 |
|  | red | 0.174 | 0.051 | **0.004** | 0.432 | 0.069 | 0.349 |
| 12° temporal | cyan | 0.517 | 0.172 | 0.055 | 0.392 | 0.143 | 0.580 |
|  | red | 0.224 | 0.094 | **0.026** | 0.529 | 0.218 | 0.585 |
